# Orthopaedics Practice in a Rural Hospital During the COVID-19 Pandemic: A Descriptive Cross-Sectional Study

**DOI:** 10.31729/jnma.5543

**Published:** 2020-11-30

**Authors:** Mangal Rawal, Tufan Singh Kathayet, Poojan Kumar Rokaya, Abhishek Kumar Thakur, Smritee Mahat, Bishnu Dutta Acharya, Dhan Bahadur Karki, Sujata Ojha

**Affiliations:** 1 Department of Orthopedic and Trauma, Karnali Academy of Health Sciences, Jumla, Nepal; 2 Department of Pathology, Institute of Medicine, Maharajgunj, Kathmandu, Nepal

**Keywords:** *COVID 19*, *elective cases*, *orthopaedic and trauma*

## Abstract

**Introduction:**

The coronavirus disease 2019 pandemic is one of the biggest public health challenges that we have faced, and has had a significant impact on the delivery of essential healthcare worldwide. Trauma and orthopaedic surgery was one of the most severely affected specialties as all non-emergency surgical cases were cancelled. With the possibility of future peak of corona virus, knowing the impact of Covid on orthopaedics would enable us to manage trauma patient in an effective manner and will help us to resume normal schedule of the trauma care.

**Methods:**

This is a descriptive cross-sectional study. We studied the details of total number of patient at Orthopaedic and trauma outpatient, inpatient and operation theatre of Karnali Academy of Health Sciences from 14th March 2020 to 16 September 2020 as compared with the patient data from the same time period in 2019.

**Results:**

There were 2288 patients during the non-Covid period who visited to the orthopaedic outpatient in comparison to 1618 during Covid period. Only 5 implant removals were performed in comparison to 50 removals (90% reduction) in elective cases. But the number of surgical cases was almost similar (204 vs 207 respectively). Total number of hospital stay had significantly reduced in the non-COVID period (average 4 days vs 6 days). There were 181 in-patient admissions during the COVID period while 241 admission during the non-COVID period.

**Conclusions:**

There is reduction in number of orthopaedic and trauma cases during COVID 19 with marked reduction in number of elective cases. Alternative methods of treatment like telemedicine and small scale health camps at peripheral districts should be conducted to treat non-urgent orthopaedic conditions during lockdown.

## INTRODUCTION

A novel corona virus was originated in Wuhan city of china and rapidly spread all over the world by March 2020.^1^ The coronavirus disease 2019 (COVID-19) pandemic is one of the biggest public health challenge that we have faced, and has had a significant impact on the delivery of essential healthcare worldwide.^2^

Trauma and orthopaedic (T&O) surgery was one of the most severely affected specialties as all non-emergency surgical cases were cancelled, outpatient consultations were reconfigured, in-patient admissions were minimized, and staff were redeployed to medical and intensive care specialties to help treat COVID-19 patients.^3^ It has caused substantial disruption of health care services including orthopaedic and trauma.^4^ Majority of patients at Karnali Academy of Health Sciences (KAHS) are related to orthopaedics and trauma. So, evaluation of orthopaedic and trauma services in our institute may provide holistic concept on clinical services in our institute.

This study aims to study the pattern of trauma care during pandemic in comparison to the non Covid period and thus provide better insight for the preparation regarding trauma care in a day to come.

## METHODS

This is a descriptive cross-sectional study done at the department of Orthopaedic and Trauma at Karnali Academy of Health Sciences. Ethical approval was taken from institutional review committee (ref no 43) of our institute. We studied the details of total number of patient at Orthopaedic and trauma outpatient, inpatient and operation theatre of Karnali academy of health sciences from 14^th^ March 2020 to 16 September 2020 as compared with the patient data from the same time period in 2019. All patients attending our hospital during the abovementioned duration during COVID and Non COVID period were included in the study.

The data regarding demography, clinical profile and treatment undergone including surgeries were collected from the hospital records. The sample size was calculated as below:

$$\begin{array}{ll} \text{Sample size (n)} & \text{= }{\text{Z}}^{\text{2}}\times \text{p}\times \text{(1}-\text{p)}/{\text{e}}^{\text{2}}\\ & \text{= }{\left(1.96\right)}^{\text{2}}\times 0.5\times (1-0.5)/{(\text{0.05)}}^{\text{2}}\\ & \text{= }384 \end{array}$$

Where,

n = required sample size
Z = 1.96 at 95% Confidence Interval (CI)
p = population proportion, 0.5
e = margin of error, 5%

Therefore, the calculated sample size was 384. However, to increase the scientific validity, the more number of samples were studied. Convenience sampling was done.

Data entry was done in MS excel and analysed using SPSS 16 version. Frequencies and percentages were used for descriptive analysis.

## RESULTS

Total numbers of patient visiting three different wards (outpatient, inpatient and operation theatre) of orthopaedic department were analysed (Table 1).

**Table 1:** Total Number of OPD cases During COVID and Non COVID period.

Period	Sex		Total	Fracture n (%)
	Male	Female		
Non-COVID Period	2288	2001	4289	Fracture 691 (16.1)
COVID Period	1618	1442	3060	Fracture 827 (27)

Table 1 shows that the total number of patients visiting to out-patient department has significantly reduced during COVID period as compared to Non COVID period. There are 2288 patient during six Non COVID month and 1618 cases during six COVID month (29.2% reduction in total patient) in outpatient department of orthopaedic and trauma. Proportion of male and female patient coming to OPD during both periods is almost same (2288 male during Non COVID Period and 1618 male patient during COVID Period). Reduction in number of patients during COVID period is probably because of strict lockdown all over the country. Cessation of elective services and lockdown are the main causes of reduction of number of patient in out-patient department. However, despite of lockdown, proportion of fracture cases out of total case increased from 691 cases (16.1%) during non COVID period to 827 (27%) during COVID period. This is because of reduction of total number of non-urgent cases in outpatient department.

The total number of procedures done during COVID is lower than non COVID (Table 2). Every surgeries performed in orthopaedics were urgent and semi urgent. The number of implant removal done for united fractures were reduced from 50 during non COVID period to 5 during COVID period. Surgeries for elective cases were deferred during the lockdown period. There is 90% reduction in elective cases during corona period.

**Table 2:** Cases in Operation Theatre.

Period	Male	Female	Total surgeries excluding implant removal for united fracture	Implant removal
Non COVID period	187	6	204	50
COVID period	167	45	207	5

However the total number of urgent and semi urgent cases which underwent surgery increased during COVID period comparing to that of Non COVID period. This can probably be explained by children staying at home during lockdown, getting more time to play and greater chances of getting injured.

The total number of hospital stay has reduced in non COVID period (average 4 days Vs. 6 days) (Table 3). There are 309 admission during Non COVID period and 249 admissions during COVID period (25% reduction) in inpatient admissions. There are 165 upper limb fractures during COVID period and 111 during Non COVID period (32.7% reduction) while incidence of lower limb fracture found to be more in Covid period.

**Table 3:** Cases admitted and discharged form Orthopaedic Ward.

	Male	Female	Total	Upper limb fracture	Lower limb fracture	Average Hospital Stay(days)
Non Covid	241	69	309	165	42	6 days (0-28)
Covid	181	68	249	111	65	4 days (0-19 days)

## DISCUSSION

Current COVID pandemic was unprecedented, so no one was able to predict the consequences of such a crisis on Orthopaedic and trauma patients. It was expected that there would be greater fall in trauma cases during lockdown as because population would be undertaking fewer risky activities and there would be less motor vehicle accidents. Many authors have mentioned about the definitive change in aetiology of trauma during pandemic. They have even hypothesized that there would be less number of work related and road traffic injuries because of lockdown. But instead our study showed that there is mild rise in trauma cases. This can partially be explained by increase in activity level of children in household as they were not going to school during lockdown. Increasing number of alcohol related fall injuries during lockdown also explains mild rise in trauma cases during lockdown. Health posts and local clinics were almost closed during lockdown period and referral to tertiary care hospital has been increased during this period. There is growing interests of researchers on observational studies to understand factors, challenges and response to this pandemic among orthopaedic services throughout the world.^6–9^

Karnali Academy of Health Sciences is the largest tertiary care centre and is a referral hospital for more than 6 districts of Karnali Province. It's very difficult to conclude the status of orthopaedic care during COVID pandemic in Nepal as there is no any comparative studies published on impact of orthopaedic and trauma services in our country till now. There was strict lockdown throughout the nation for long, so there is clear-cut reduction in number of patients visiting in orthopaedic department of Karnali Academy of Health Sciences. As in many centres throughout Nepal, elective cases were halted in our centre. Various strategies were adapted by orthopaedic surgeon to combat this pandemic all over the world.^10–12^ Many countries have adopted the policy of cessation of elective and non-urgent orthopaedic surgeries during pandemic.^13–17^ There is 90% reduction in elective cases (implant removal) in our setup which correspond to the article published by various authors on Covid 19 and orthopaedic services.^4^ Minimization in hospitalisation of patient and clinical appointment, effective continuation of emergency attendances and proper guiding of staffs and resources deployment for the sustenance of emergency service was done as in other studies.

Single centre study with short study period is the main limitation of this study. Multicentre study with large number of patient population over the longer duration may predict the exact impact of COVID 19 on orthopaedic and trauma services.

## CONCLUSIONS

There is reduction in number of orthopaedic and trauma cases during COVID 19 with marked reduction in number of elective cases. Alternative methods of treatment like telemedicine and small scale health camps at peripheral districts should be conducted to treat non-urgent orthopaedic conditions during lockdown.
